# Supplementary material for: A compendium of chromatin contact maps reflecting regulation by chromatin remodelers in budding yeast
Source: Nat Commun. 2021 Nov 4;12:6380. doi: 10.1038/s41467-021-26629-6 (PMC8569116; doi:10.1038/s41467-021-26629-6)
Supplement: Supplementary file 1 — Supplementary information [file 41467_2021_26629_MOESM1_ESM.pdf]

Supplementary information for

A compendium of chromatin contact maps reflecting regulation by chromatin remodelers in budding yeast.

Supplementary Figures 1-9

Supplementary Tables 1-4

Hyelim Jo[1], Taemook Kim[1], Yujin Chun[1], Inkyung Jung[1], Daeyoup Lee[1,*]

[1]Department of Biological Sciences, Korea Advanced Institute of Science and Technology, 291 Daehak-ro, Yuseong-gu, Daejeon 34141, Republic of Korea

Phone: +82-42-350-2623

Fax: +82-42-350-2610

E-mail: dylee@kaist.ac.kr

Supplementary Figure 1

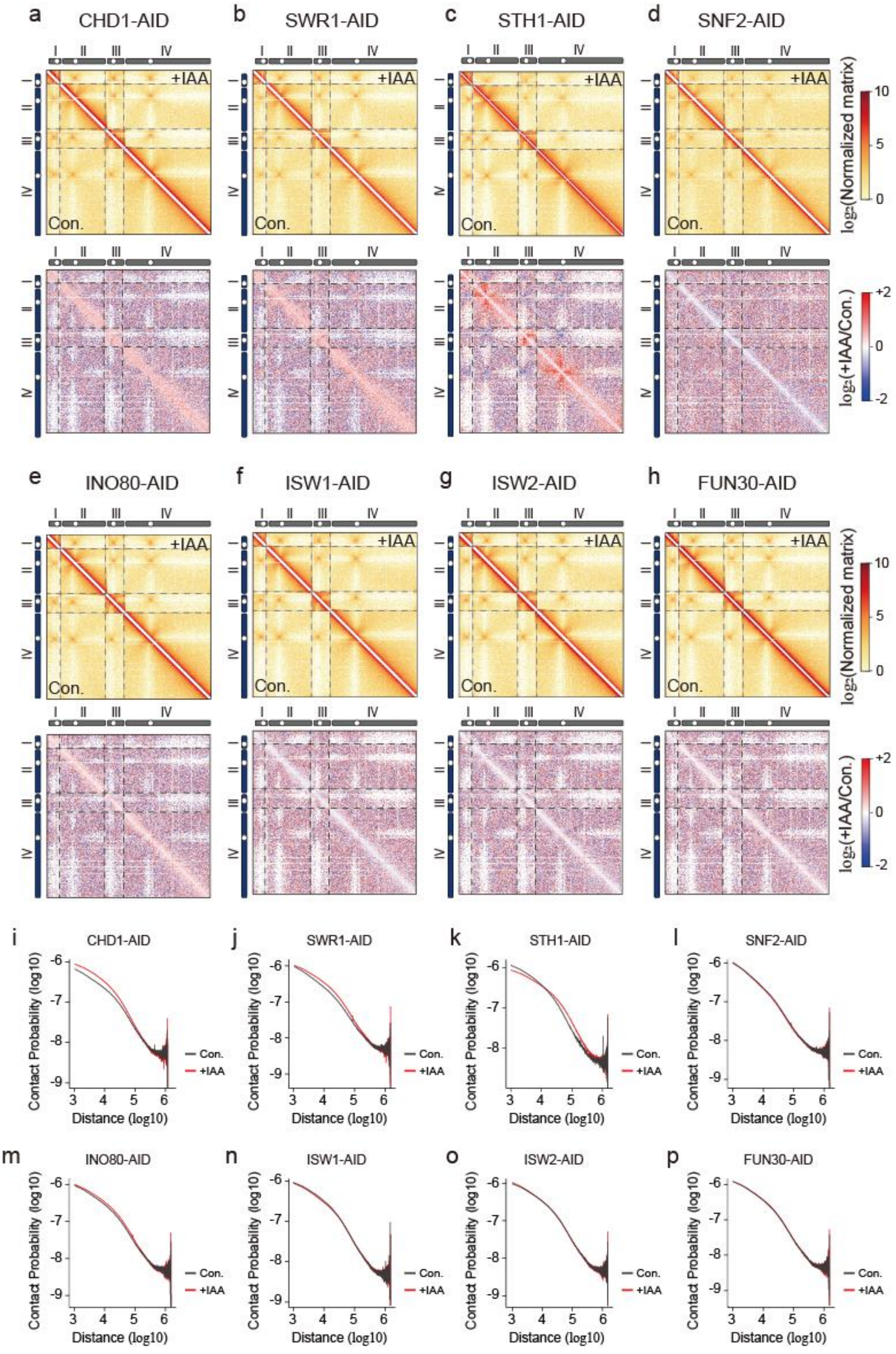

Supplementary Figure 1. The studied chromatin remodelers can regulate the 3D architecture of chromatin (Async.).

(a-h) Heatmaps (5-kb resolution) showing the chromosomal interactions from chromosome 1(I) to chromosome 4 (VI) for each chromatin remodeler *AID* strain. IAA (indole-3-acetic acid, auxin; 0.5 mM) was used to deplete each chromatin remodeler, and the same volume of ethanol was used as a control. The top panels show the ICE-normalized matrix of the control (bottom left, 'Con.') and knockdown (top right, '+IAA') conditions, and the bottom panel shows the log_2 ratio between control and knockdown samples. (i-p) Average contact probability (CP) according to genomic distance of *AID* strains.

Supplementary Figure 2

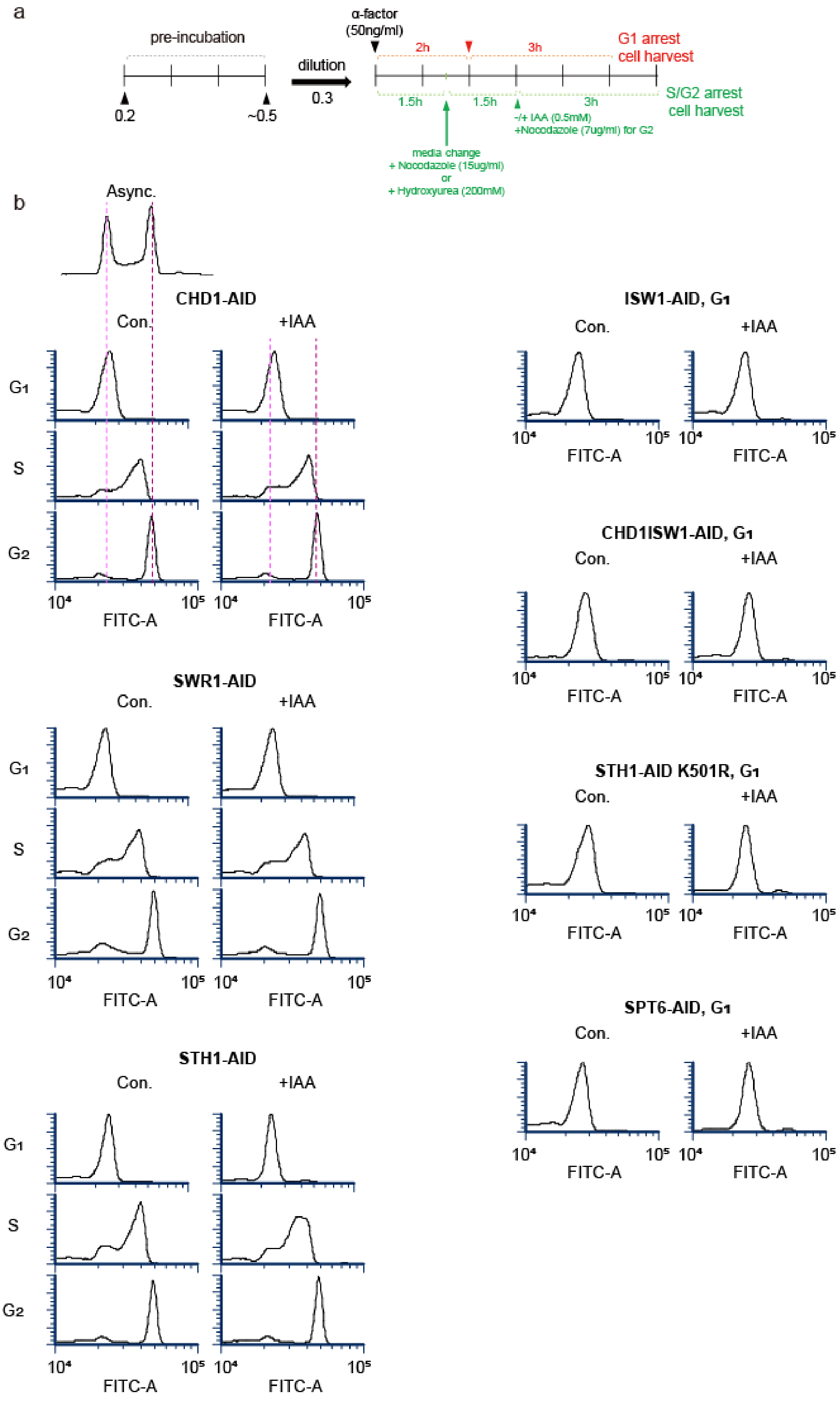

Supplementary Figure 2. Flow cytometric analysis of cell cycle distributions for AID strains.

(a) Experimental scheme showing how we obtained the synchronized yeast cells used for *in situ* Hi-C. The red text indicates the process used to collect cells arrested at G_1, while the green text indicates those used to collect cells arrested at S and G_2. The same volume of ethanol was used for the -IAA samples. (b) Flow cytometric analysis for confirmation of cell cycle arrest at G_1, S, and G_2. The DNA contents (e.g., 1N, 2N) were quantified using SYTOX green (FITC) signals. The x-axis indicates the level of DNA content and the y-axis indicates the cell count. The singlet cells were used for cell cycle analysis and gating strategies are provided source data file.

Supplementary Figure 3

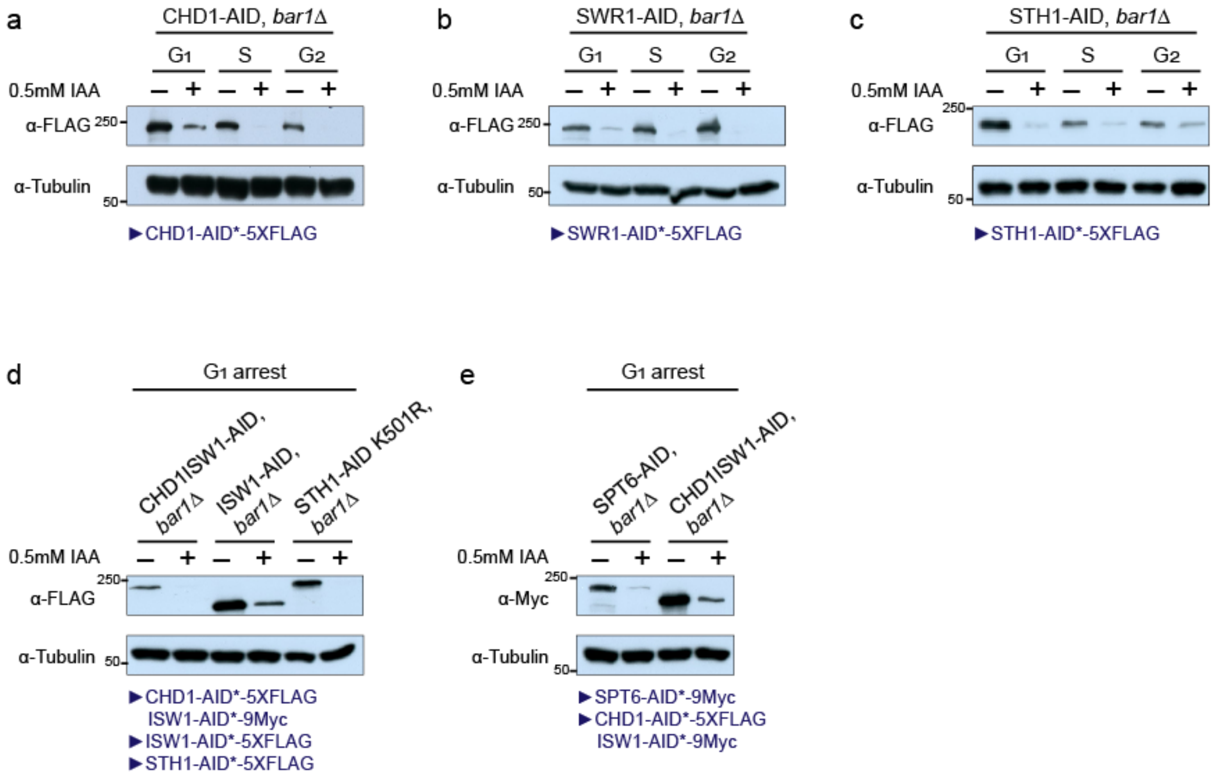

Supplementary Figure 3. Chromatin remodeler proteins are degraded upon IAA treatment after cell cycle synchronization.

(a-c) Chd1p, Swr1p, and Sth1p were degraded after 3 hrs of IAA treatment under G_1, S, and G_2 phase arrest conditions. α-FLAG antibody (Sigma; F7425, 1:5000) was used to detect Chd1p, Swr1p, or Sth1p. (d) Chd1p, Isw1p, and Sth1p were blotted using α-FLAG antibody after G1 arrest. (e) Spt6p and Isw1p were detected by α-Myc antibody (Cell Signaling; 2276S, 1:3000) after G1 arrest. α-Tubulin antibody (Abcam; ab6061, 1:5000) was used for a loading control (a-e). (a-e) are representative figures of two biologically independent experiments.

Supplementary Figure 4

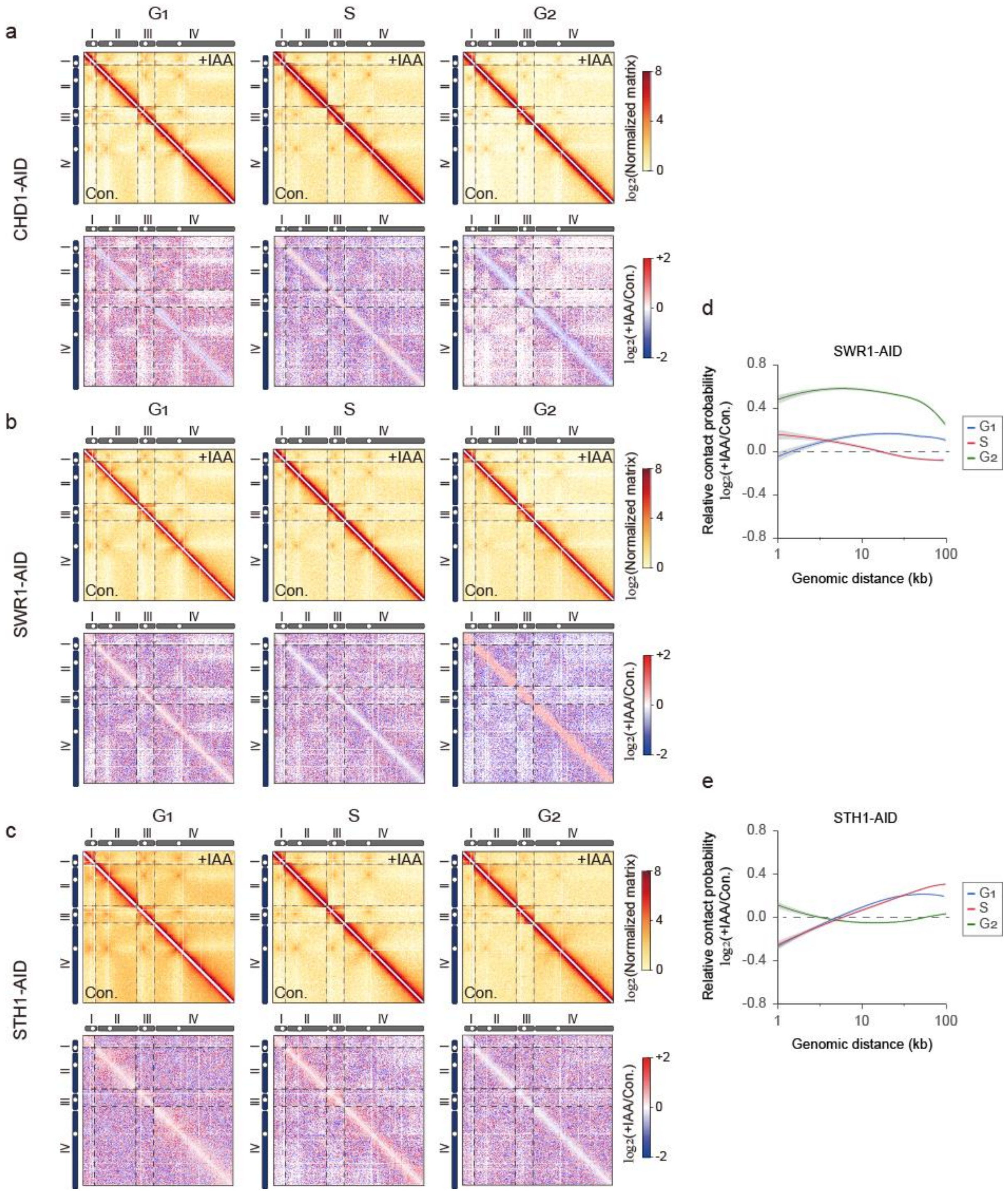

Supplementary Figure 4. Chd1, Swr1p, and Sth1p dynamically regulate the 3D genome structure throughout the cell cycle.

(a-c) Heatmaps (5-kb resolution) showing the chromosomal interactions from chromosome 1(I) to chromosome 4(VI) for CHD1-AID, SWR1-AID, and STH1-AID strains at G_1, S, and G_2 phase. The top

panels show the ICE-normalized matrices of the control (bottom left, 'Con.') and knockdown (top right, '+IAA') conditions, and the bottom panel shows the log_2 ratio between control and knockdown samples. (d-e) Log_2 ratio of the average contact probability (CP) along genomic distance between control (Con.) and IAA-treated (+IAA) samples at G_1, S, and G_2 phases for SWR1-AID and STH1-AID strains. The gray shadow indicates the confidence interval around smooth (se).

Supplementary Figure 5

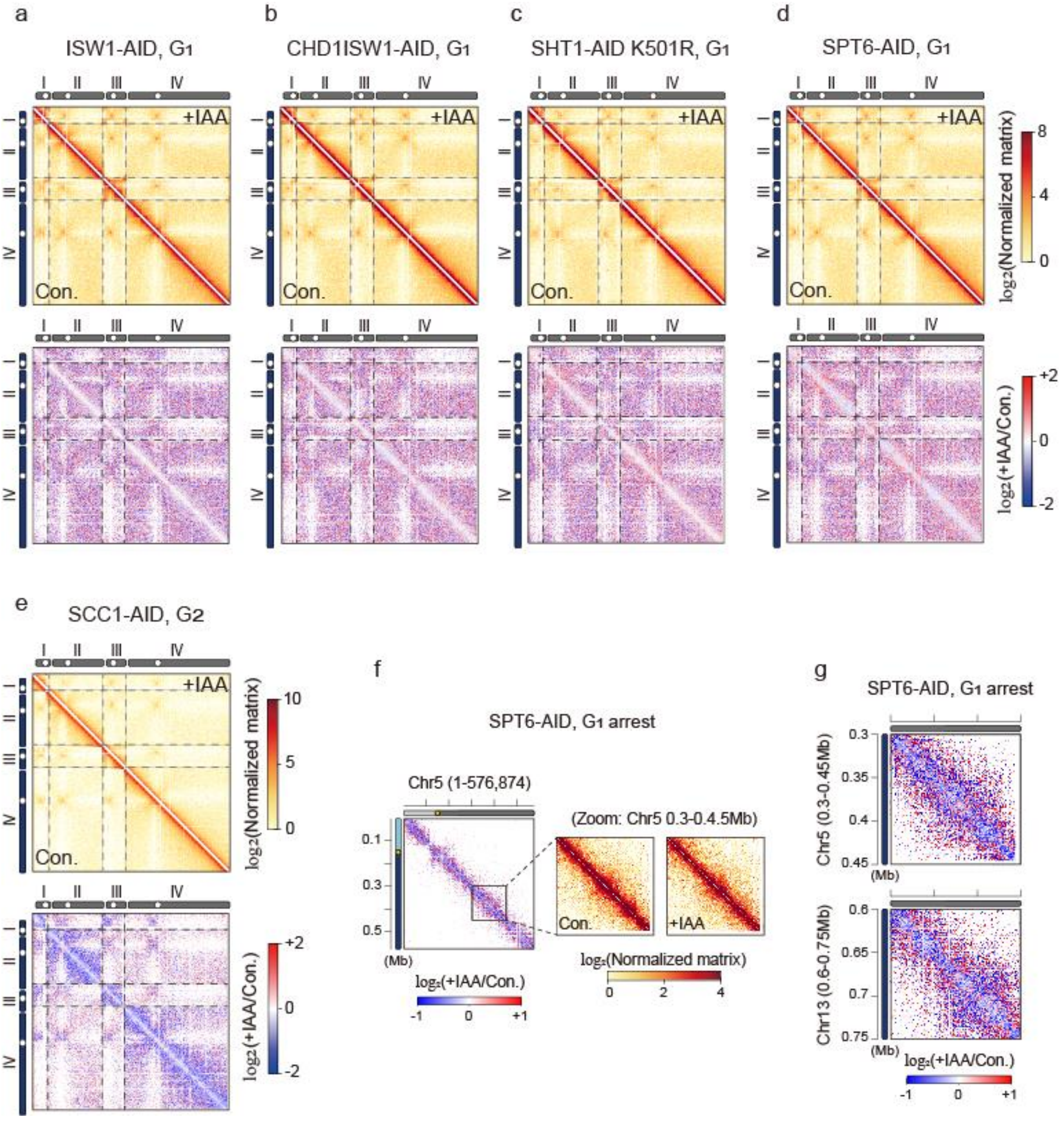

Supplementary Figure 5. The 3D structure of the genome is controlled by several factors.

(a-e) Heatmaps (5-kb resolution) showing the chromosomal interactions from chromosome 1(I) to chromosome 4(VI) for ISW1-AID (a), CHD1ISW1-AID (b), STH1-AID K501R (c), and STP6-AID (d) strains at G_1 phase. The GSM2417297 dataset was used to perform the same analysis of SCC1-AID strain at G_2 phase (e). The top panels show the ICE-normalized matrices of the control (bottom left, 'Con.') and knockdown (top right, '+IAA') conditions, and the bottom panel shows the log_2 ratio between control and knockdown samples. (f) Heatmap (1-kb resolution) showing the chromosomal interactions

within chromosome 5 for SPT6-AID strain. The left panel shows the log_2 ratio between control and knockdown samples. The right two panels show zoom-in matrices of chromosome 5 (0.3-0.45 Mb) for the control ('Con.') and knockdown ('+IAA') conditions. (g) Log_2 ratio interaction maps (1-kb resolution) between control and knockdown samples for chromosome 5 (0.3-0.45 Mb; upper panel) and chromosome 13 (0.6-0.75 Mb; lower panel).

Supplementary Figure 6

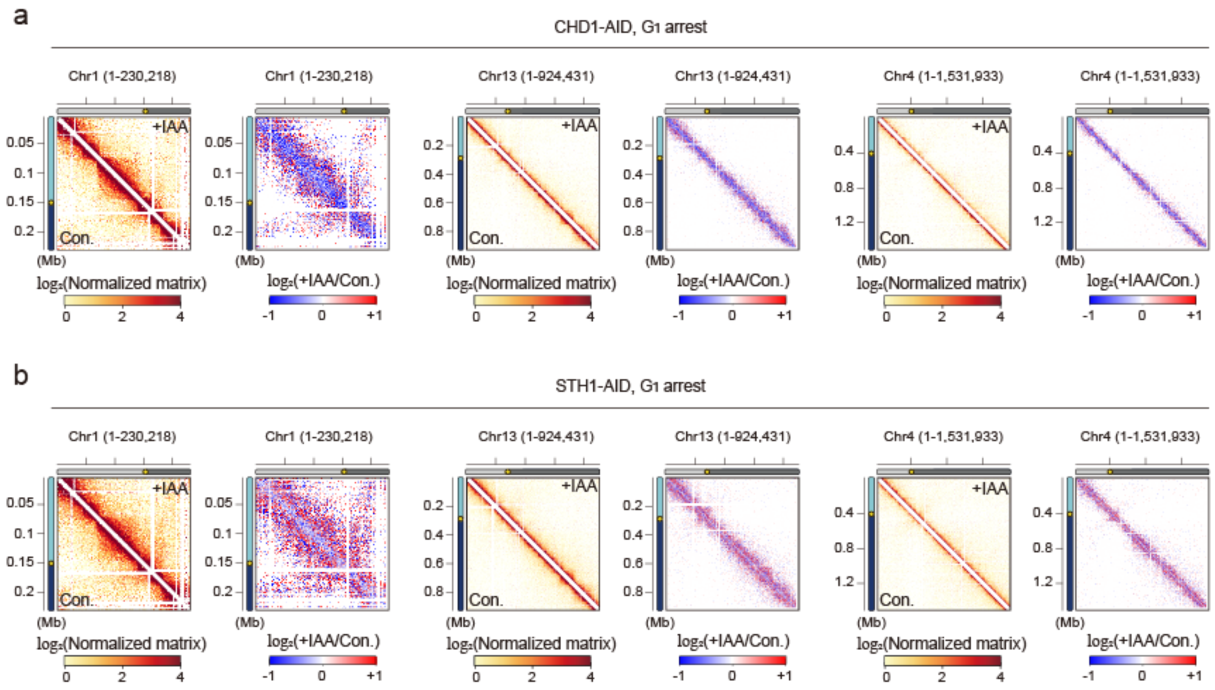

Supplementary Figure 6. The chromatin remodelers show common functions in 3D genome organization.

(a) Interaction maps (1-kb resolution) for chromosomes 1, 13, and 4 in CHD1-AID strain at G_1 phase. The left panel shows the ICE-normalized matrix and the right panel shows the $\log_2$ ratio between control and knockdown samples. (b) Same as in (a) but for STH1-AID strain at G_1 phase.

Supplementary Figure 7

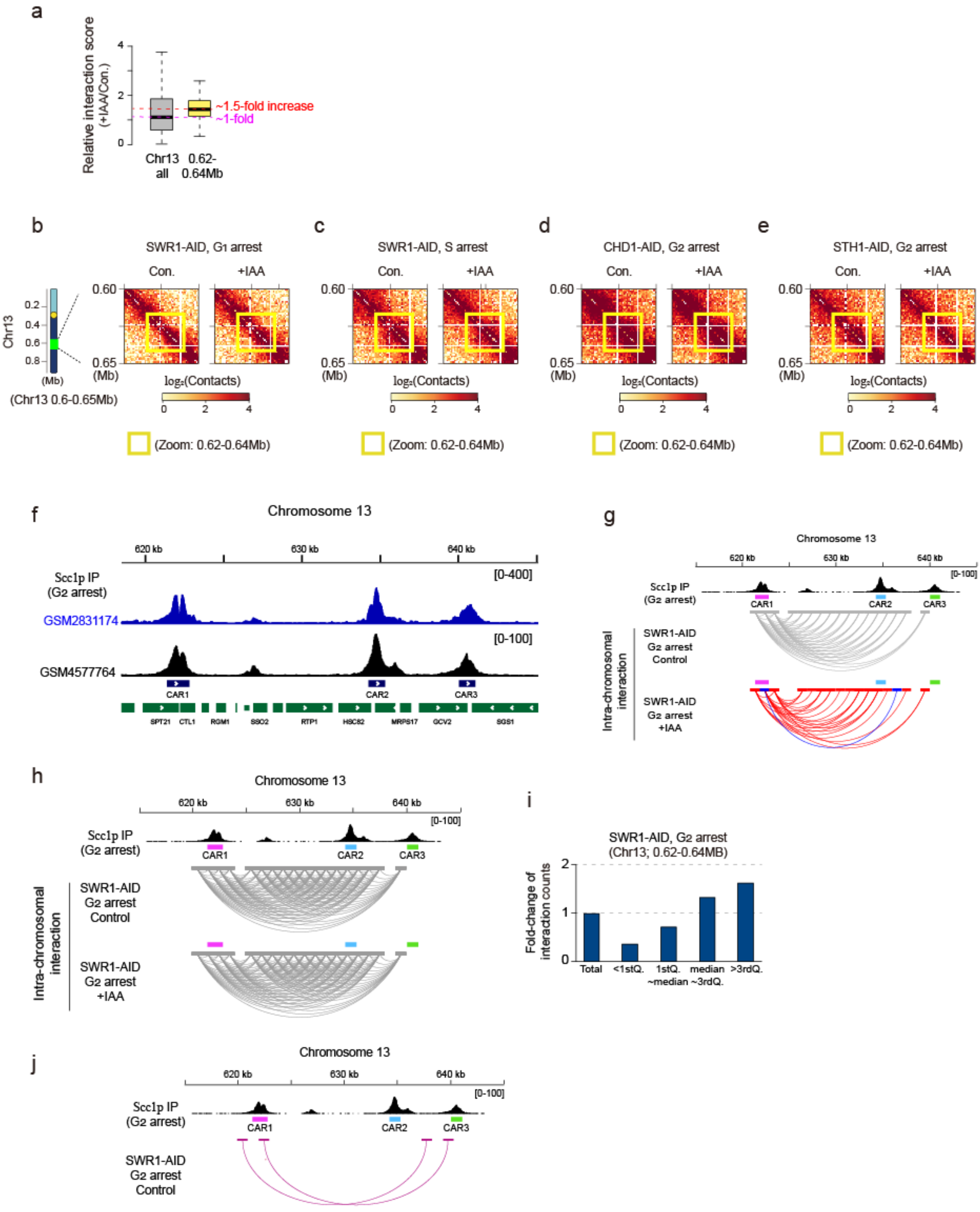

Supplementary Figure 7. Swr1p is important for modulation of loop- like structures on chromosome 13 during G_2 phase.

(a) Boxplot showing the fold-change of interaction score before and after IAA treatment in chromosome 13 (n= 157460) or only in 0.64-0.64Mb region (n= 170) on chromosome 13. The average fold-change indicated by using the value of median. Boxplot show median; box limits, upper and lower quartiles; whiskers. (b, c) Zoom-in log_2 ratio interaction maps of chromosome 13 (0.6-0.65 Mb region) in SWR1-AID strain at G_1 and S phases. Schematic representations including genomic distances and positions are displayed on the left side of (b) and the yellow dots indicate the point-centromere of the chromosome. (d, e) Zoom-in log_2 ratio interaction maps of chromosome 13 (0.6-0.65 Mb region) in CHD1-AID (d) and STH1-AID (e) strains at G_2 phase. (f) IGV data visualizing Scc1p IP signals and their peak on chromosome 13 under nocodazole-induced G_2/M arrest. Datasets GSM283114 and GSM4577764 were analyzed. (g) (top) IGV data for Scc1p (GSM4577764) on chromosome 13 at G_2 phase. Pink, cyan, and yellow-green boxes indicate cohesin-associate regions (CARs) 1, 2 and 3. (bottom) Arc plot displaying intra-chromosomal interactions with CAR1 loci. The gray lines indicate whole intra-chromosomal contacts in control. The red lines indicated the increased contacts after IAA treatment while the blue lines indicated the decreased contacts upon IAA treatment. (h) IGV data indicated cohesion bounded-CAR and arc plot for whole intra-chromosomal interactions in control and +IAA sample. (i) The fold-change of intra-chromosomal interactions before and after IAA treatment in chromosome 13 or only in 0.64-0.64Mb region on chromosome 13. Total 168 contacts were divided into four groups (25%) by interaction score; (1) <1st Quantile, (2) >1st but <2nd Quantile (median), (3) >2nd but <3rd Quantile and (4) >3rd Quantile. (j) IGV data indicated cohesion bounded-CAR and arc plot showing the 14kb-distance intra-chromosomal interactions which were increased over 2-fold in +IAA.

Supplementary Figure 8

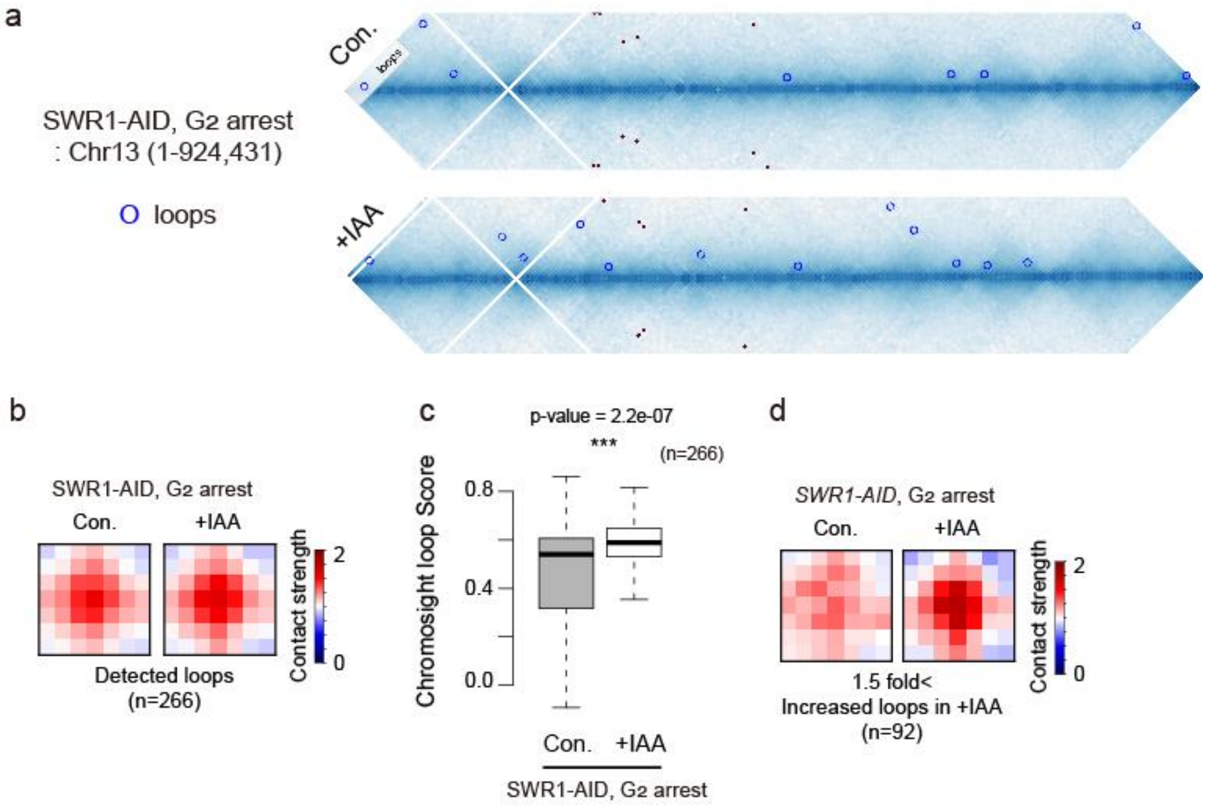

Supplementary Figure 8. Swr1p is involved in modulation of loop- like structures on all 16 chromosomes during G_2 phase.

(a) The interaction map of chromosome 13 for the control ('Con.') and knockdown ('+IAA') conditions in SWR1-AID strain at G_2 phase. The blue circles indicate loop positions detected by Chromosight. (b) the aggregate plot for detected loop position in control and +IAA samples from SWR1-AID strain at G_2 phase. (c) Boxplot for detected loops (n=266). The p-values were calculated by a one-sided Wilcoxon rank-sum test (*** means p-value<0.001). Boxplot show median; box limits, upper and lower quartiles; whiskers. (d) Same as in (b) but showing loop positions that exhibited increases of >1.5-fold upon IAA treatment.

Supplementary Figure 9

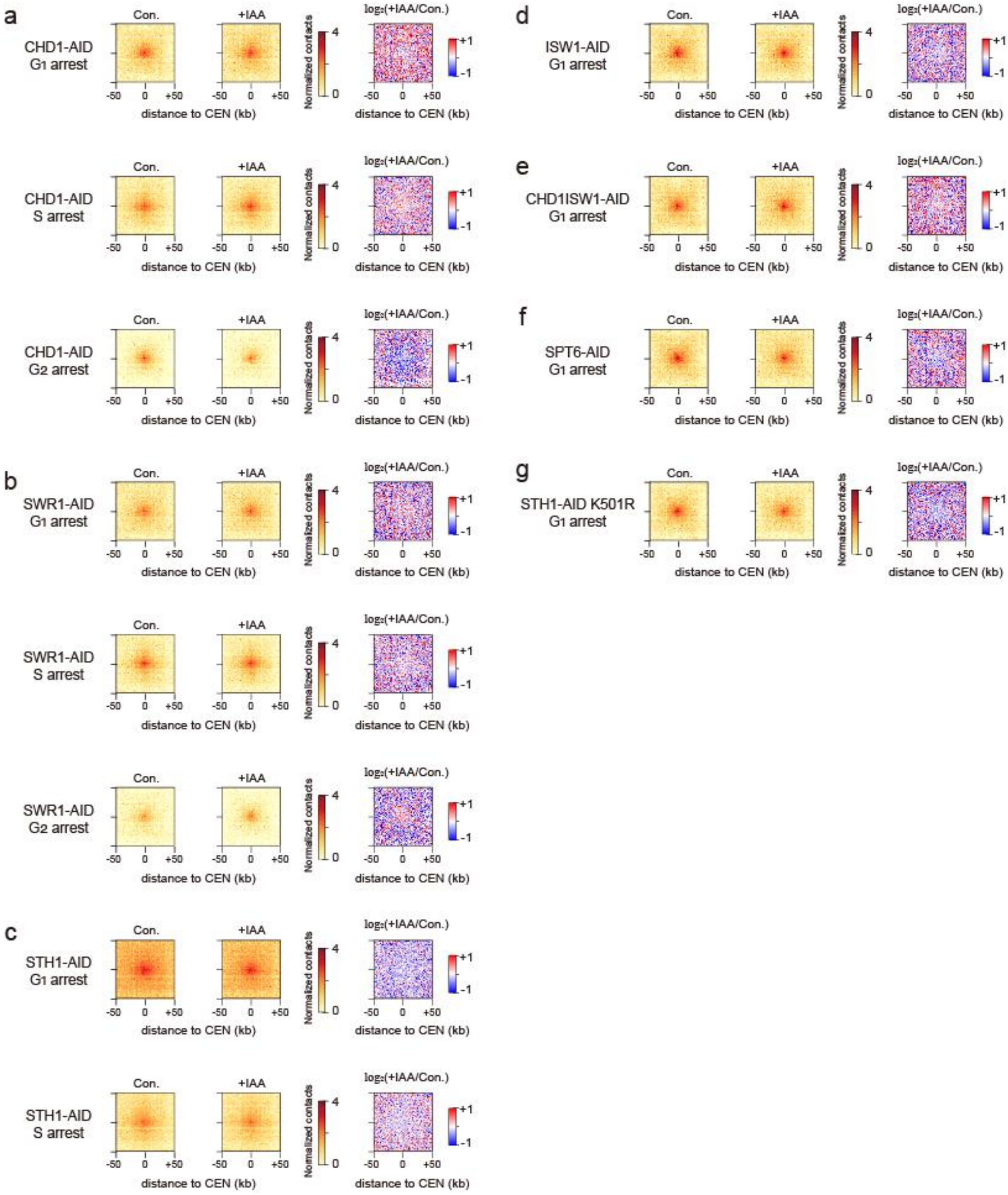

Supplementary Figure 9. Chd1p and Swr1p affect centromere clustering during G_2 phase.

(a-g) Average matrices with 1-kb resolution showing inter CEN-CEN interactions (left panels) and their $\log_2$ ratios (right panels).

Supplementary Table 1. Yeast strain information

U2721, genetic background:DF5, MATa, his3-Δ200, leu2-3,2-112, lys2-801, trp1-1(am), URA3::TIR-9Myc	Morawska et al., 2013	
CHD1-IAA*-FLAG, genetic background:DF5, paternal strain: U2721, his3-Δ200, leu2-3,2-112, lys2-801, trp1-1(am), URA3::TIR-9Myc, CHD1-44AID9Flag::hphNT	Klein-Brill et al., 2019	NF188
INO80-IAA*-FLAG, genetic background:DF5, paternal strain: U2721, his3-Δ200, leu2-3,2-112, lys2-801, trp1-1(am), URA3::TIR-9Myc, INO80-44AID9Flag::hphNT	Klein-Brill et al., 2019	NF191
SWR1-IAA*-FLAG, genetic background:DF5, paternal strain: U2721, his3-Δ200, leu2-3,2-112, lys2-801, trp1-1(am), URA3::TIR-9Myc, SWR1-44AID9Flag::hphNT	Klein-Brill et al., 2019	NF193
SNF2-IAA*-FLAG, genetic background:DF5, paternal strain: U2721, his3-Δ200, leu2-3,2-112, lys2-801, trp1-1(am), URA3::TIR-9Myc, SNF2-44AID9Flag::hphNT	Klein-Brill et al., 2019	NF196
STH1-IAA*-FLAG, genetic background:DF5, paternal strain: U2721, his3-Δ200, leu2-3,2-112, lys2-801, trp1-1(am), URA3::TIR-9Myc, STH1-44AID9Flag::hphNT	Klein-Brill et al., 2019	NF198
FUN30-IAA*-FLAG, genetic background:DF5, paternal strain: U2721, his3-Δ200, leu2-3,2-112, lys2-801, trp1-1(am), URA3::TIR-9Myc, FUN30-44AID9Flag::hphNT	Klein-Brill et al., 2019	NF200
ISW1-IAA*-FLAG, genetic background:DF5, paternal strain: U2721, his3-Δ200, leu2-3,2-112, lys2-801, trp1-1(am), URA3::TIR-9Myc, ISW1-44AID9Flag::hphNT	Klein-Brill et al., 2019	NF202
ISW2-IAA*-FLAG, genetic background:DF5, paternal strain: U2721, his3-Δ200, leu2-3,2-112, lys2-801, trp1-1(am), URA3::TIR-9Myc, ISW2-44AID9Flag::hphNT	Klein-Brill et al., 2019	NF204
SPT6-IAA*-MYC, genetic background:DF5, paternal strain: U2721, his3-Δ200, leu2-3,2-112, lys2-801, trp1-1(am), URA3::TIR-9Myc, SPT6-AID9Myc:: kanmx	This paper	SC1188
SPT16-IAA*-MYC, genetic background:DF5, paternal strain: U2721, his3-Δ200, leu2-3,2-112, lys2-801, trp1-1(am), URA3::TIR-9Myc, SPT16-AID9Myc::kanmx	This paper	SC1178
SCC1-IAA*-FLAG, genetic background:DF5, paternal strain: U2721, his3-Δ200, leu2-3,2-112, lys2-801, trp1-1(am), URA3::TIR-9Myc, SCC1-AID6Flag::hphNT	This paper	SC1260
SCC2-IAA*-FLAG, genetic background:DF5, paternal strain: U2721, his3-Δ200, leu2-3,2-112, lys2-801, trp1-1(am), URA3::TIR-9Myc, SCC2-AID6Flag::hphNT	This paper	SC1262
bar1Δ CHD1-IAA*-FLAG<br>his3-Δ200, leu2-3,2-112, lys2-801, trp1-1(am), URA3::TIR-9Myc, CHD1-44AID9Flag::hphNT, *bar1Δ*::natmx	This paper	SC1377
bar1Δ SWR1-IAA*-FLAG<br>his3-Δ200, leu2-3,2-112, lys2-801, trp1-1(am), URA3::TIR-9Myc, SWR1-44AID9Flag::hphNT, *bar1Δ*::natmx	This paper	SC1365
bar1Δ STH1-IAA*-FLAG<br>his3-Δ200, leu2-3,2-112, lys2-801, trp1-1(am), URA3::TIR-9Myc, STH1-44AID9Flag::hphNT, *bar1Δ*::natmx	This paper	SC1191
bar1Δ ISW1-IAA*-FLAG<br>his3-Δ200, leu2-3,2-112, lys2-801, trp1-1(am), URA3::TIR-9Myc, ISW1-44AID9Flag::hphNT *bar1Δ*::natmx	This paper	SC1381
bar1Δ CHD1-IAA*-FLAG, ISW1-IAA*-MYG<br>his3-Δ200, leu2-3,2-112, lys2-801, trp1-1(am), URA3::TIR-9Myc, CHD1-44AID9Flag::hphNT, ISW1-44AID9Myc::kanmx *bar1Δ*::natmx	This paper	SC1367
bar1Δ SPT6-IAA*-MYC, his3-Δ200, leu2-3,2-112, lys2-801, trp1-1(am), URA3::TIR-9Myc, SPT6-AID9Myc:: kanmx *bar1Δ*::natmx	This paper	SC1372
bar1Δ STH1-IAA*-FLAG STH1-K501R, his3-Δ200, leu2-3,2-112, lys2-801, trp1-1(am), URA3::TIR-9Myc, STH1-44AID9Flag::hphNT, STH1-K501R-V5::Leu, *bar1Δ*::natmx	This paper	SC1374

Supplementary Table 2. Primer sequences (5’-to-3’)

Primer name	Sequence
ISW1_degron_F	GTTGGTAGCAGAGAAAATTCCGGAAAACGAAACCACTCATcgtacgctgcaggtcgac
ISW1_degron_R	AGGATATATTAAAAAAAATCGAAATATAAAAAAAGAAGGTatcgatgaattcgagctcg
ISW1_check_F	GAG GGA TTG TAT TGG ATG ACG C
SPT6_degron_F	aaaatctaacagtagtaagaatagaatgaacaactaccgt cgtacgctgcaggtcgac
SPT6_degron_R	ATAATAAAATTAATAATAACAATGGACACTACATACGCAT atcgatgaattcgagctcg
SPT6_check_F	GTT GAA CCA CGA TAA CCC TGG TT
bar1_deletion_F1	CGCCTAAAATCATACCAAAATAAAAAGAGTGTCTAGAAGGGTCATATACGGATCCCCGGGTTAATTAA
bar1_deletion_R1	ATATTTGATATTTATATGCTATAAAGAAATTGTACTCCAGATTTCGAATTCGAGCTCGTTTAAAC
bar1_deletion_check_F	CTATTCTGAA ACACACCACA
Kan_R	CTGCAGCGAGGAGCCGTAAT
XmaI_Sth1_pro_F	TTTCCCGGGTCGCGCTTGCTCTAAACTGTG
bamHI_Sth1_PK_R	TTTGGATCCTTACGTAGAATCGAGACCGAGGAGAGGGTTAGGGATAGGCTTACCCGAAGAGTGTTCCTTGAACCATT
STH1_K501R_F	ATG GGT TTA GGT CGA ACC ATC CAA TCT
STH1_K501R_R	AGA TTG GAT GGT TCG ACC TAA ACC CAT

Supplementary Table 3. Valid interaction reads

(Async.)

	CHD1_Con.	CHD1+IAA	SWR1_Con.	SWR1+IAA	STH1_Con.	STH1+IAA	SNF2_Con.	SNF2+IAA
valid_interaction	20580057	32878071	27768746	15896822	31394612	36857666	19100291	21426071
valid_interaction rmdup	17478203	28556212	23968520	14044357	28071361	32023686	17575645	19703338
trans_interaction	11201012	16017466	14949996	7781844	15011180	15554521	11537821	13084828
cis_interaction	6277191	12538746	9018524	6262513	13060181	16469165	6037824	6618510
cis_shortRange	2972073	6061752	4910145	3250745	6976477	6998120	3307636	3710013
cis_longRange	3305118	6476994	4108379	3011768	6083704	9471045	2730188	2908497

	INO80_Con.	INO80+IAA	ISW1_Con.	ISW1+IAA	ISW2_Con.	ISW2+IAA	FUN30_Con.	FUN30+IAA
valid_interaction	41303929	45335035	34406459	29466392	35324695	32272865	48996204	42659997
valid_interaction rmdup	35834828	38430553	29630013	25839899	30430458	27903035	42437915	37238036
trans_interaction	21462670	21098198	16473497	14661685	17217409	15567171	22983492	19606454
cis_interaction	14372158	17332355	13156516	11178214	13213049	12335864	19454423	17631582
cis_shortRange	7450491	9014819	6649226	5488905	6614695	6323794	10631389	9681317
cis_longRange	6921667	8317536	6507290	5689309	6598354	6012070	8823034	7950265

	SCC1_Con.	SCC1+IAA	SCC2_Con.	SCC2+IAA	SPT6_Con.	SPT6+IAA	SPT16_Con.	SPT16+IAA
valid_interaction	17819654	13922566	20602966	15539428	23453625	24445474	21235825	16426358
valid_interaction rmdup	16390186	12745964	18707107	14146630	21335718	22038877	19347216	14924838
trans_interaction	10882541	9473222	12488980	11065631	13617331	14338977	12468823	9911734
cis_interaction	5507645	3272742	6218127	3080999	7718387	7699900	6878393	5013104
cis_shortRange	2948208	1766471	3449384	1580653	4374018	3926276	4006947	2349843
cis_longRange	2559437	1506271	2768743	1500346	3344369	3773624	2871446	2663261

(Cell cycle arrest)

	CHD1-G1_Con.	CHD1-G1+IAA	CHD1-S_Con.	CHD1-S+IAA	CHD1-G2_Con.	CHD1-G2+IAA
valid_interaction	15563530	13228313	11331119	19334256	17786875	15514701
valid_interaction rmdup	**13782902**	**11905761**	**10215941**	**17560643**	**16146847**	**14077774**
trans_interaction	7157801	7171420	5902241	9519283	4578149	4837785
cis_interaction	6625101	4734341	4313700	8041360	11568698	9239989
cis_shortRange	4439538	2782879	2378788	4679238	7690271	6283530
cis_longRange	2185563	1951462	1934912	3362122	3878427	2956459

	SWR1-G1_Con.	SWR1-G1+IAA	SWR1-S_Con.	SWR1-S+IAA	SWR1-G2_Con.	SWR1-G2+IAA
valid_interaction	12799895	14057476	17486422	19988187	17447728	22244354
valid_interaction rmdup	**11398883**	**12229590**	**15681710**	**17797584**	**15621308**	**19942966**
trans_interaction	7013197	7301535	8715963	9780358	8159886	7336394
cis_interaction	4385686	4928055	6965747	8017226	7461422	12606572
cis_shortRange	2636118	2895470	3948494	4734488	4594080	8141227
cis_longRange	1749568	2032585	3017253	3282738	2867342	4465345

	STH1-G1_Con.	STH1-G1+IAA	STH1-S_Con.	STH1-S+IAA	STH1-G2_Con.	STH1-G2+IAA
valid_interaction	19980703	19397564	12541689	15905893	12411424	13767848
valid_interaction rmdup	**18425598**	**17780998**	**11502170**	**14442674**	**11440620**	**12668323**
trans_interaction	12018615	11433833	6360076	7815520	5933012	6618022
cis_interaction	6406983	6347165	5142094	6627154	5507608	6050301
cis_shortRange	3656256	3335050	2919773	3388595	2813787	3083823
cis_longRange	2750727	3012115	2222321	3238559	2693821	2966478

	ISW1-G1_Con.	ISW1-G1+IAA	CHD1ISW1-G1_Con.	CHD1ISW1-G1+IAA	K501R-G1_Con.	K501R-G1+IAA
valid_interaction	20923059	20387880	17604889	17696871	18726127	24507545
valid_interaction rmdup	**19086464**	**18284023**	**15862852**	**15911409**	**16886216**	**21849682**
trans_interaction	10107521	9613703	8226389	8724548	8762569	11585761
cis_interaction	8978943	8670320	7636463	7186861	8123647	10263921
cis_shortRange	5801451	5609410	5068752	4536941	5340343	6333553
cis_longRange	3177492	3060910	2567711	2649920	2783304	3930368

	SPT6-G1_Con.	SPT6-G1+IAA	G2_Con.	SCC1-G2+IAA
valid_interaction	21143246	21391307	19709964	5167494
valid_interaction rmdup	**19084811**	**19330808**	19005791	5066725
trans_interaction	9611369	10633343	10146972	3046650
cis_interaction	9473442	8697465	8858819	2020075
cis_shortRange	6421618	5417487	4284986	1183155
cis_longRange	3051824	3279978	4573833	836920

Supplementary Table 4. Replication test (SCC and Pearson correlation value)
Shown are the stratum-adjusted correlation coefficient (SCC) and Pearson product-moment correlation coefficient between replicates

- SCC value option: -v 18 -m 150000, whole chromosome average, 1-kb resolution matrix input

Sample_Name (Rep1-Rep2)	SCC_value	Pearson
CHD1_G1_Con.	0.98758513	0.999
CHD1_G1_IAA	0.98538099	0.999
CHD1_S_Con.	0.97750346	0.987
CHD1_S_IAA	0.98322703	0.996
CHD1_G2_Con.	0.99067601	0.999
CHD1_G2_IAA	0.98737691	0.999
SWR1_G1_Con.	0.980904	0.998
SWR1_G1_IAA	0.98487592	0.997
SWR1_S_Con.	0.98643656	0.999
SWR1_S_IAA	0.98699355	0.999
SWR1_G2_Con.	0.9805686	0.999
SWR1_G2_IAA	0.99116411	0.999
STH1_G1_Con.	0.98771416	0.998
STH1_G1_IAA	0.9885877	0.999
STH1_S_Con.	0.9848053	0.998
STH1_S_IAA	0.98720728	0.997
STH1_G2_Con.	0.9835362	0.999
STH1_G2_IAA	0.98539059	0.998
ISW1_G1_Con.	0.99163226	0.998
ISW1_G1_IAA	0.99084796	0.996
CHD1ISW1_G1_Con.	0.98969335	0.992
CHD1ISW1_G1_IAA	0.98944276	0.999
STH1K501R_G1_Con.	0.98870504	0.999
STH1K501R_G1_IAA	0.99310418	0.998
SPT6_G1_Con.	0.99201977	0.996
SPT6_G1_IAA	0.99063258	0.994
**Control_sample (Sample1/2)**	**SCC_value**	
SWR1_G1/S_Con1	0.82314842	
SWR1_G1/G2_Con1	0.88650709	
SWR1_G2/S_Con1	0.85857394	
