# Supplementary material for: A compendium of chromatin contact maps reflecting regulation by chromatin remodelers in budding yeast
Source: Nat Commun. 2021 Nov 4;12:6380. doi: 10.1038/s41467-021-26629-6 (PMC8569116; doi:10.1038/s41467-021-26629-6)
Supplement: Supplementary file 3 — Reporting Summary [file 41467_2021_26629_MOESM3_ESM.pdf]

nature research

Corresponding author(s): Daeyoup Lee

Last updated by author(s): Sep 30, 2021

Reporting Summary

Nature Research wishes to improve the reproducibility of the work that we publish. This form provides structure for consistency and transparency in reporting. For further information on Nature Research policies, see our Editorial Policies and the Editorial Policy Checklist.

Statistics

For all statistical analyses, confirm that the following items are present in the figure legend, table legend, main text, or Methods section.

n/a	Confirmed	
[ ]	[x]	The exact sample size (*n*) for each experimental group/condition, given as a discrete number and unit of measurement
[ ]	[x]	A statement on whether measurements were taken from distinct samples or whether the same sample was measured repeatedly
[ ]	[x]	The statistical test(s) used AND whether they are one- or two-sided<br>*Only common tests should be described solely by name; describe more complex techniques in the Methods section.*
[x]	[ ]	A description of all covariates tested
[ ]	[x]	A description of any assumptions or corrections, such as tests of normality and adjustment for multiple comparisons
[ ]	[x]	A full description of the statistical parameters including central tendency (e.g. means) or other basic estimates (e.g. regression coefficient) AND variation (e.g. standard deviation) or associated estimates of uncertainty (e.g. confidence intervals)
[ ]	[x]	For null hypothesis testing, the test statistic (e.g. *F*, *t*, *r*) with confidence intervals, effect sizes, degrees of freedom and *P* value noted<br>*Give P values as exact values whenever suitable.*
[x]	[ ]	For Bayesian analysis, information on the choice of priors and Markov chain Monte Carlo settings
[x]	[ ]	For hierarchical and complex designs, identification of the appropriate level for tests and full reporting of outcomes
[ ]	[x]	Estimates of effect sizes (e.g. Cohen's *d*, Pearson's *r*), indicating how they were calculated

Our web collection on statistics for biologists contains articles on many of the points above.

Software and code

Policy information about availability of computer code

Data collection	Standard Illumina NovaSeq 6000 and HiSeqX ten procedures and software for in situ Hi-C data sequencing.<br>BD FACSDiva™ Software for BD LSRFortessa (FACS).
Data analysis	-For in situ Hi-C and ChIP-seq analyses we used following tools:<br>HiC-Pro (v2.11.1) (http://nservant.github.io/HiC-Pro/)<br>HiCPlotter (v0.8.1) (https://github.com/kcakdemir/HiCPlotter)<br>HiCExplorer (v3.4.3) (https://github.com/deeptools/HiCExplorer/)<br>HiCcompare (v1.8.0) (https://github.com/dozmorovlab/HiCcompare)<br>IGV (v2.10.2) (https://software.broadinstitute.org/software/igv/)<br>Chromosight (v1.4.1) (https://github.com/koszullab/chromosight)<br>HiCRep.py (v0.0.6) (https://github.com/dejunlin/hicrep)<br>Bowtie2 (v2.3.4.1) (http://bowtie-bio.sourceforge.net/bowtie2/index.shtml)<br>HOMER (v4.10.4) (http://homer.ucsd.edu/homer/)<br>-For statistical analyses and plot, we used R (R studio, R version 3.6.3)<br>-For FACS data chart generation, we used FCS Express 7 Research Edition (https://denovosoftware.com/)

For manuscripts utilizing custom algorithms or software that are central to the research but not yet described in published literature, software must be made available to editors and reviewers. We strongly encourage code deposition in a community repository (e.g. GitHub). See the Nature Research guidelines for submitting code & software for further information.

Data

Policy information about availability of data

All manuscripts must include a data availability statement. This statement should provide the following information, where applicable:

- Accession codes, unique identifiers, or web links for publicly available datasets
- A list of figures that have associated raw data
- A description of any restrictions on data availability

Raw .fastq and processed data of in situ Hi-C are accessible via the SRA repository [GSE158336, https://www.ncbi.nlm.nih.gov/geo/query/acc.cgi?acc=GSE158336]. The ChIP-seq data for Scc1p at G2 phase were obtained from [GSM283117443, https://www.ncbi.nlm.nih.gov/geo/query/acc.cgi?acc=GSM283117443] and [GSM457776429, https://www.ncbi.nlm.nih.gov/geo/query/acc.cgi?acc=GSM457776429].
The Hi-C data for Scc1p at G2 phase were obtained from [GSM2417297, https://www.ncbi.nlm.nih.gov/geo/query/acc.cgi?acc=GSM2417297].

Field-specific reporting

Please select the one below that is the best fit for your research. If you are not sure, read the appropriate sections before making your selection.

- [x] Life sciences
- [ ] Behavioural & social sciences
- [ ] Ecological, evolutionary & environmental sciences

For a reference copy of the document with all sections, see nature.com/documents/nr-reporting-summary-flat.pdf

Life sciences study design

All studies must disclose on these points even when the disclosure is negative.

Sample size	High-throughput genome-wide experiments were performed in duplicate and all of duplicates show minimal variation (Supplementary Table 4). The reproducibility between duplicates were sufficient to determine differences between samples.
Data exclusions	No experiments were excluded from analysis.
Replication	We used biological replicates for in situ Hi-C experiments and all attempts were successful (Supplementary Table 4).
Randomization	Experiments were not randomized since randomization was not relevant to our study.
Blinding	Researchers were not blinded during experiments and data analysis. Blinding was not required for our study.

Reporting for specific materials, systems and methods

We require information from authors about some types of materials, experimental systems and methods used in many studies. Here, indicate whether each material, system or method listed is relevant to your study. If you are not sure if a list item applies to your research, read the appropriate section before selecting a response.

Materials & experimental systems

n/a	Involved in the study	
	x	Antibodies
x		Eukaryotic cell lines
x		Palaeontology and archaeology
x		Animals and other organisms
x		Human research participants
x		Clinical data
x		Dual use research of concern

Methods

n/a	Involved in the study	
x		ChIP-seq
	x	Flow cytometry
x		MRI-based neuroimaging

Antibodies

Antibodies used	anti-Flag (Sigma, #7425, 1:5000), anti-Myc (Cell signaling, #2276S, 1:3000) for stain check and anti-tubulin(abcam, #ab6061, 1:5000) for loading control. anti-Rabbit (Jackson ImmunoResearch, #111-035-003, 1:20000), anti-Mouse (Jackson ImmunoResearch, #115-035-003, 1:20000) and anti-Rat (Jackson ImmunoResearch, #112-035-003, 1:20000) were used for secondary antibodies for anti-Flag, anti-Myc and anti-tubulin respectively.
Validation	For those commercial antibodies, the validation statements are provided on the manufacturer's websites, and were used as guidelines.

Flow Cytometry

Plots

Confirm that:

- [x] The axis labels state the marker and fluorochrome used (e.g. CD4-FITC).
- [x] The axis scales are clearly visible. Include numbers along axes only for bottom left plot of group (a 'group' is an analysis of identical markers).
- [ ] All plots are contour plots with outliers or pseudocolor plots.
- [ ] A numerical value for number of cells or percentage (with statistics) is provided.

Methodology

Sample preparation	For cell cycle analysis, yeast cells fixed with ethanol. After rehydration and 2.5 µM Sytox Green (Invitrogen S7020) staining steps, sonicate cells (30%, 1 sec ON/ 1 sec OFF) and stored at 4℃ until using analyzer.
Instrument	BD LSRFortessa cell analyzer
Software	The .fcs data were manipulated with FCS Express software.
Cell population abundance	Total 0.1milion yeast cells collected at G1, S, G2 phase respectively. The 98% of cells passed after singlet gating.
Gating strategy	We only removed doublet of yeast cells. The gating strategy is provided in the Source-data file.

- [x] Tick this box to confirm that a figure exemplifying the gating strategy is provided in the Supplementary Information.
